# Dr. Inman on the Progress of Rational Medicine

**Published:** 1858-04

**Authors:** 


### DR. INMAN ON THE PROGRESS OF A RATIONAL MEDICINE.*

Dr. Inman, in the work under discussion, marches in the vanguard of the hygienics. His book has a curious title, for which he frankly apologises. His writing has a meaning which makes up for all misfortunes of title. His views on the progress of medicine are thus expounded:

"When the profession shall have recognised the principles we have attempted to lay down; when they begin to look with jealousy at every dose of calomel, antimony, or digitalis that they order; when they take for a starting-point the axiom that 'disease implies debility,' and 'that everything which weakens a patient must impede his restoration to health;' when bleeding shall have been dismissed to the limbo of scientific fallacies, to be recalled only by some medical judge of supreme knowledge; when blisters become the rifle-shot of the experienced hunter, who never fires without a definite aim, rather than the grape, canister, or the shrapnel-shot of the artillerist, who fires them comparatively at random; when purgatives are not considered panaceas, and salivation is not a refuge for the destitute of ideas; when active treatment gives way to scientific, and the general health is considered superior to the apparent health of any one organ; when medicines

* The Phenomena of Spinal Irritation, and other Functional Diseases of the Nervous System, explained, and a Rational Plan of Treatment deduced. By THOMAS INMAN, M.D.Lond. London: Churchill. 1858.

are considered as means to an end, and not as so many doses of bottled comfort; when the doctor recognises in recovery the result of a natural process rather than the imbibition of so many ounces of physic; when there is a thorough knowledge of what medicine can do, as well as what it cannot; when a discrimination is made between the effects of a disease and the effect of presumed remedies; when the natural history of each complaint becomes more generally known, not only will the science of medicine be fixed upon a firm basis, but it will command a confidence that it has never yet fully deserved.

"At present, the tendency of medical authors is to hold a magnifying glass before particular organs, to discriminate between the minutest phases of their complaints, and to discuss the best plans of this or that symptom. What is wanted is a broad and comprehensive classification, in which life, health, vitality, and nutrition will form the genera, and diseases the species only. The question the physician will then propose to himself will be, how shall I restore the patient to health? not how shall I attack the complaint? When an organ has gone wrong, or an inflammation has set itself up, instead of punishing the former, and knocking down the latter, the system will be helped to put the one right and to get over the other. A difficulty will be 'tided over' instead of being crushed. Rapidity of cure rather than an ultimate result will be the test of successful theory; and oh! conclusion most disastrous, the more scientific the physician the smaller will be his emoluments."

